# 388. Epidemiologic and Microbiologic Characteristics of Hospital-acquired Infections in Patients with COVID-19 at Intensive Care Unit, Mexico City

**DOI:** 10.1093/ofid/ofab466.589

**Published:** 2021-12-04

**Authors:** Paulo Castañeda-Méndez, Maria Lorena Cabrera-Ruiz, Armando Barragán-Reyes, Esperanza Aleman Aguilar, Brenda Aceves Sanchez, Maria Fernanda Ruiz Salgado, Maria Jose Ribe Viesca, Brenda Alexia Velez Ortiz, Javier Reyes Mar, Daniel Aguilar-Zapata, Claudia Yanet Ruiz Hernandez, Gloria Santillan Lopez, Eva Juarez Hernandez, Alejandra Jimena Garcia Martinez, Luis E Soto-Ramirez, Lili Frias

**Affiliations:** 1 Hospital Medica Sur / Hospital San Angel Inn Universidad, Mexico City, Distrito Federal, Mexico; 2 Medica Sur, Mexico City, Distrito Federal, Mexico; 3 MEDICA SUR, Mexico City, Distrito Federal, Mexico; 4 Hospital Medica Sur, Mexico City, Distrito Federal, Mexico

## Abstract

**Background:**

Patients with severe SARS-CoV-2 infection are at high risk of complications due to the intensive care unit stay. Hospital-acquired infections (HAI) are one of the most common complication and cause of death in this group of patients, it is important to know the epidemiology and microbiology of this hospital-acquired infections in order to begin to the patients a proper empirical treatment. We describe the epidemiologic and microbiologic characteristics of HAI in patients with COVID-19 hospitalized at intensive care unit (ICU) in a tertiary level private hospital in Mexico City.

**Methods:**

From April to December 2020, data from all HAIs in patients with severe pneumonia due to SARS-CoV-2 infection with mechanical ventilation at ICU were obtained. The type of infection, microorganisms and antimicrobial susceptibility patterns were determined.

**Results:**

A total of 61 episodes of HAIs were obtained, the most common was ventilator associated pneumonia (VAP) in 52.4% (n=32) followed by urinary tract infection (UTI) 34.4%(n=21) and bloodstream infection (BSI) 9.84% (n=6). Only two episodes corresponded to *C. difficile* associated diarrhea. We identified 82 different microorganisms, the most frequent cause of VAP was *P. aeruginosa* 22% (10/45) followed by *K. pneumoniae* 20% (9/45); for UTI, *E. coli* 28.5% (6/21), and *S. marcescens* 19% (4/21); for BSI the most frequent microorganism was *S. aureus* 28.5 (2/7). Regarding the antimicrobial susceptibility patters the most common were Extended Spectrum Beta-Lactamase (ESBL) Gram-negative rods followed by Methicillin-resistant *Staphylococcus aureus*.

**Conclusion:**

In patients with severe COVID-19 hospitalized in the ICU the most frequent HAIs were VAP and UTI caused by P. aeruginosa and E. coli respectively. ESBL enterobacteriaceae was the most common resistant pattern identifed in the bacterial isolations in our series.

**Disclosures:**

**All Authors**: No reported disclosures

